# Supplementary material for: Fingerprinting of Volatile Organic Compounds in Old and Commercial Apple Cultivars by HS-SPME GC/GC-ToF-MS
Source: Int J Mol Sci. 2024 Dec 16;25(24):13478. doi: 10.3390/ijms252413478 (PMC11676995; doi:10.3390/ijms252413478)
Supplement: Supplementary file 1 [file ijms-25-13478-s001.zip › Suplementary.pdf]

Table S2 The cluster analysis based on 12 components generated with 70 compounds present in the apples of the commercial cultivars. The Ward method combined with Euclidean distance was applied.

	**Cluster analysis (Factor coordinates of cases) Linking distance = 15.5068**												
	**Belonging to cluster**	**Comp. 1**	**Comp. 2**	**Comp. 3**	**Comp. 4**	**Comp. 5**	**Comp. 6**	**Comp. 7**	**Comp. 8**	**Comp. 9**	**Comp. 10**	**Comp. 11**	**Comp. 12**
**Lobo 7**	1	-17.1596	0.15467	3.013	-3.62322	-1.64797	-1.7471	-1.09936	1.36457	1.77518	-0.48056	0.47011	-0.70053
**Melrose 4**	2	1.3771	3.38531	1.8786	-0.47283	1.99583	-1.27234	-0.86683	-0.31504	-0.26376	-0.49761	-0.84966	-0.37184
**Melrose 6**	2	1.3004	6.34181	1.29481	-0.82754	2.75195	-2.59068	-2.07425	-0.86342	-1.34707	-2.31621	-0.90427	-1.18497
**Melrose 8**	2	0.2242	7.88685	4.23916	-1.97705	4.52641	-2.0575	-1.65654	-0.48096	-1.31143	3.09049	0.34736	0.70053
**Gala 1**	3	-0.3798	1.16096	-2.87084	-0.59299	-0.97734	0.24548	0.89022	-0.21625	0.8777	1.31743	-0.20526	-1.35023
**Gala 2**	3	-1.0992	1.19734	-3.09216	2.69588	-0.94395	-0.48978	1.2512	0.9207	0.8496	2.78117	0.62276	0.33445
**Gala 3**	3	0.0006	1.281	-2.17088	0.59677	-2.37911	0.58673	-0.19754	-0.01817	0.83585	2.16962	1.35726	1.27016
**Gala 4**	3	-0.6822	1.85486	-3.01857	1.6853	-0.43814	-2.47955	1.27333	0.92634	-0.29493	1.29928	0.45812	-1.31979
**Gala 5**	3	0.2859	0.87888	-2.77937	3.15212	-3.29808	-3.20376	-1.15081	1.58676	-0.14252	-0.94356	-0.35551	3.3896
**Gala 6**	3	0.2799	0.81075	-3.97035	2.82312	-0.95478	-2.93964	-1.7685	0.19465	0.82798	-1.42404	-0.40023	0.43522
**Gala 7**	3	0.0632	1.88733	-4.15416	2.53783	-0.57014	-2.58364	-0.67853	-0.23794	0.89614	-1.00802	-0.54687	-1.1235
**Red Prince 3**	4	1.5847	2.32459	0.27674	-0.16492	-1.85812	0.82557	0.10528	-0.57518	-0.35274	-0.45667	0.51478	0.40711
**Red Prince 5**	4	3.5643	4.38144	2.52742	-3.98504	-3.41318	0.82868	3.83094	-0.73554	1.07662	-2.40615	0.68677	-0.19333
**Red Prince 6**	4	2.0776	2.0918	2.10733	-2.73011	-1.38872	-1.05341	4.18678	0.98669	0.25776	-1.03364	1.93661	-0.8441
**Red Prince 7**	4	1.1584	3.24222	-0.58435	1.01305	-1.82628	0.5235	0.2126	-0.44087	-0.51498	-1.12825	-0.13679	0.38413
**Red Prince 8**	4	1.6126	4.44499	3.02448	-1.14008	-2.23634	2.47819	-0.29625	-1.56848	0.9637	2.27748	-1.54233	2.23841
**Ligol 1**	5	0.9401	-3.00896	2.96174	1.99486	-2.55302	-1.08068	0.38245	2.1869	-5.22346	1.29784	-0.96791	-2.71429
**Ligol 2**	5	0.0983	-2.50003	4.47307	1.49492	-1.38785	1.29147	-1.61917	-1.21925	0.94967	1.31409	-2.475	-0.49071
**Ligol 3**	5	0.9316	-2.98533	0.38984	2.10956	1.70229	-1.55903	1.34411	-0.87835	1.20428	1.07315	0.30404	-0.25031
**Ligol 4**	5	1.2633	-2.58689	3.18909	2.44912	0.65847	-0.03559	0.81847	0.47182	0.23602	-0.40455	0.84185	0.76456
**Ligol 5**	5	0.2509	-0.2582	3.82943	1.24942	-3.91982	3.82592	-1.51078	3.10802	-0.46379	-1.02101	-2.32233	0.08879
**Ligol 6**	5	0.985	-1.221	-0.43124	1.36022	0.05281	-1.05379	0.39626	-0.25079	-0.88351	-0.47749	0.92982	-1.22127
**Ligol 7**	5	1.466	-6.8619	7.31313	2.72111	-0.35644	-2.84719	-0.99763	-3.28765	1.75546	-0.56178	1.9633	-0.22551
**Ligol 8**	5	1.6976	-2.3157	2.59176	1.52301	1.2491	0.30133	-0.05417	1.48286	-0.11707	-0.05252	1.84867	2.34755
**Lobo 1**	6	-8.1081	-0.67742	-1.18545	3.17941	2.41226	2.33614	4.22474	-2.70767	-3.13711	-1.3283	-1.1986	2.04598

**Lobo 5**	6	0.3349	1.2469	0.80116	2.10584	4.82629	4.32776	-1.10093	4.27238	0.6793	-1.20233	2.74459	-0.15913
**Lobo 6**	6	-0.5067	0.24423	-1.29918	2.52036	2.27511	1.77731	0.3786	-2.49821	0.81073	-0.95766	-1.50556	-0.82925
**Lobo 8**	6	-0.6845	0.08372	-0.84824	2.11114	2.40845	2.10829	-0.11161	-0.06839	0.95435	-0.46387	-0.16749	-1.71866
**Szampion 1**	7	-0.0922	-1.23327	-2.80053	-1.16494	-0.05521	1.64405	-1.06061	-0.70232	-0.2363	0.73328	1.64367	-0.26042
**Szampion 2**	7	0.9711	-1.20893	-2.56728	-0.82074	-0.45359	1.41672	-0.43799	-0.25262	1.09111	0.15045	-0.48882	-1.3418
**Szampion 3**	7	0.1929	-2.32272	-2.45348	-2.81463	0.51177	1.9731	-1.2492	-0.74903	-0.69623	-0.32861	-0.46232	0.45324
**Szampion 4**	7	2.9798	-5.87664	-0.26075	-4.87696	3.24323	-3.65321	2.37516	2.80178	1.26736	0.17437	-3.35672	1.07015
**Szampion 5**	7	0.7263	-1.39959	-1.85367	-1.71734	0.34105	2.27516	-0.49815	-0.3623	0.95119	0.5321	-0.29484	-0.81754
**Szampion 6**	7	1.6844	-3.49614	-2.64508	-4.78752	-0.94747	0.05615	-3.53131	-1.18518	-1.54866	-2.14343	0.89396	0.87181
**Szampion 7**	7	-0.2938	-3.4313	-1.99733	-3.9723	-1.1735	0.16075	-0.34472	-1.34997	-2.57406	1.65915	2.0793	-0.23224
**Szampion 8**	7	1.4756	-1.21708	-1.57304	-1.3382	0.75133	1.55392	0.19232	0.10691	1.60591	0.6119	-0.96333	-1.33421
**Szampion 9**	7	-0.5208	-2.29854	-1.3548	-2.31662	3.0727	0.11066	0.4424	0.5532	-0.7583	0.15449	-0.49913	1.88197

Table S3 Comparison of 34 representative VOC content in commercial apple cultivars. Values of content in µg/kg of fresh fruit.

VOC	Commercial apple cultivar											
	Gala		Melrose		Red Prince		Lobo		Ligol		Szampion	
**Pent-1-en-3-one**	0.56	ab	0.00	a	3.51	ab	0.00	a	0.00	a	6.04	b
**Hept-2-enal**	0.41	a	3.69	ab	3.24	ab	2.16	ab	19.57	b	11.36	b
**Pentanol**	91.21	a	48.62	a	42.76	a	86.61	ab	118.97	ab	217.45	b
**Propyl butyrate**	0.00	a	3821.08	b	0.00	a	377.61	ab	0.00	a	209.73	ab
**6-Methylhept-5-en-2-ol**	74.16	b	0.00	a	80.97	b	63.46	b	55.50	b	117.12	b
**2-Methylbutanol**	478.78	b	353.38	ab	611.60	b	250.19	ab	103.42	a	265.51	ab
**Oct-1-en-3-ol**	0.00	a	21.14	ab	23.10	ab	21.57	ab	59.80	b	44.60	b
**Benzaldehyde**	5.54	b	0.00	a	0.00	a	2.17	ab	2.50	ab	4.27	ab
**Butyl 2-methylbutyrate**	386.03	ab	712.33	b	441.27	ab	224.74	ab	0.00	a	0.00	a
**Hexyl 2-methylbutyrate**	418.35	ab	797.59	ab	926.59	b	250.18	ab	250.55	a	209.56	ab
**Heptan-2-ol**	0.00	a	0.00	a	0.00	a	45.13	b	41.45	b	0.00	a
**Hex-2-en-1-ol**	165.71	a	296.61	ab	345.37	ab	275.99	ab	480.35	b	341.52	ab
**Butyl butyrate**	746.69	ab	737.29	a	1001.08	ab	846.80	ab	1303.64	b	1069.47	ab
**Farnezen (sum of isomers)**	392.33	ab	252.10	ab	724.91	ab	394.47	ab	1257.46	b	247.98	a
**Hexyl butyrate**	355.98	a	303.95	a	845.02	ab	581.08	ab	2385.33	b	1004.69	ab
**Butanol**	1174.48	b	408.32	a	514.93	ab	530.72	ab	807.68	ab	999.14	ab
**Butyl propanoate**	477.09	ab	451.64	ab	646.99	b	174.39	ab	174.01	ab	115.14	a
**Hex-2-enal**	1146.46	a	2715.55	b	1908.11	ab	1355.83	ab	1695.80	ab	1833.39	ab
**2-Methylbutyl acetate**	1229.42	abc	6473.59	c	2379.03	bc	893.17	abc	419.61	a	463.99	ab
**Hexyl hexanoate**	478.10	a	1228.74	b	621.44	ab	638.82	ab	693.68	ab	553.47	ab
**Hexyl propanoate**	480.00	ab	583.76	b	509.56	b	196.95	ab	429.45	ab	106.31	a
**Hexyl octanoate**	67.29	ab	0.00	a	29.95	ab	0.00	a	278.86	b	0.00	a
**Butyl 2-methylpropanoate**	0.00	a	0.00	a	0.00	a	0.00	a	16.86	ab	79.85	b
**Hex-2-en-1-yl acetate**	37.04	a	401.47	ab	473.64	ab	149.77	ab	530.07	b	417.02	ab
**Propyl acetate**	421.89	ab	712.91	b	203.25	ab	45.11	ab	0.00	a	0.00	a
**Butyl acetate**	6859.28	ab	1837.24	ab	7230.45	b	1688.74	a	6550.50	ab	5735.19	ab
**Hexyl acetate**	5374.93	abc	5138.11	abc	11004.06	c	2356.35	a	8106.69	bc	4308.48	a

**Pentyl acetate**	274.23	a	617.96	ab	577.38	ab	705.26	b	600.06	ab	601.00	ab
**Octanoic acid**	20.29	b	0.00	a	0.00	a	16.44	ab	6.93	ab	8.39	ab
**Hept-3-en-6-ol**	0.00	a	0.00	a	0.00	a	47.13	ab	85.13	b	0.00	a
**2,3-Dihydrofuranone**	18.05	b	0.00	a	0.95	ab	8.91	ab	0.65	ab	1.91	ab
**Estragole**	105.32	b	33.70	ab	83.90	ab	1.66	a	42.00	ab	36.88	ab
**2-Ethylhexanol**	61.74	b	0.00	a	0.00	a	27.30	ab	19.07	ab	48.18	ab
**Hex-1-en-5-yl acetate**	108.78	b	0.00	a	0.00	a	0.00	a	0.00	a	379.14	b
